# Supplementary material for: Synthesis of Multifunctional Eu(III) Complex Doped Fe3O4/Au Nanocomposite for Dual Photo-Magnetic Hyperthermia and Fluorescence Bioimaging
Source: Molecules. 2023 Jan 11;28(2):749. doi: 10.3390/molecules28020749 (PMC9865881; doi:10.3390/molecules28020749)
Supplement: Supplementary file 1 [file molecules-28-00749-s001.zip › molecules-2147451-supplementary.pdf]

Supplementary Materials

Manuscript Title: Synthesis of multifunctional Eu(III) complex doped Fe_3O_4/Au nanocomposite for dual photo-magnetic hyperthermia and fluorescence bioimaging

Manuscript Number: Molecules-2147451

Article Type: SI: Functional Nanomaterials in Analytical and Biomedical Sciences

Corresponding author: Dr. Hoang Thi Khuyen
Tel.: +84 4 973756768; fax: + 84 4 38360705.
E-mail address: khuyenht@ims.vast.ac.vn; khuyenhtims@gmail.com

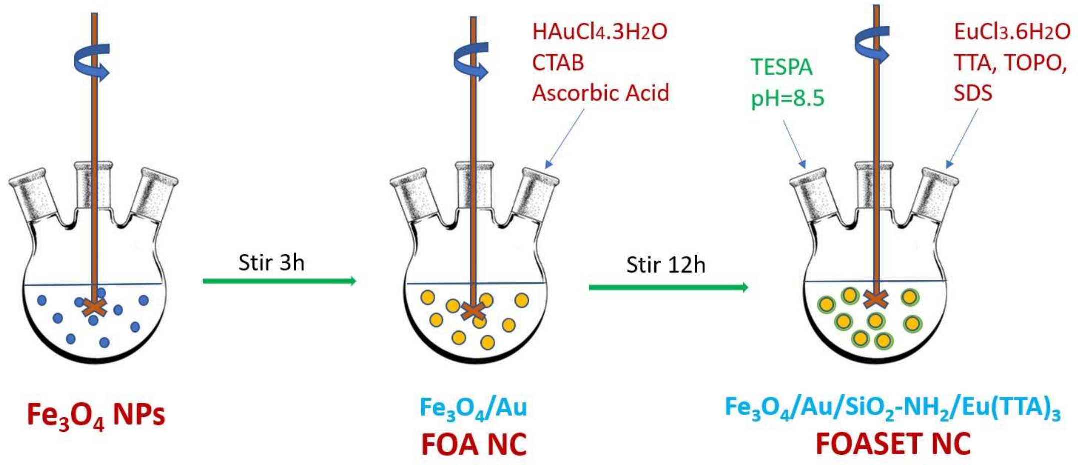

Figure S1. Steps of synthesis of FOASET NC.

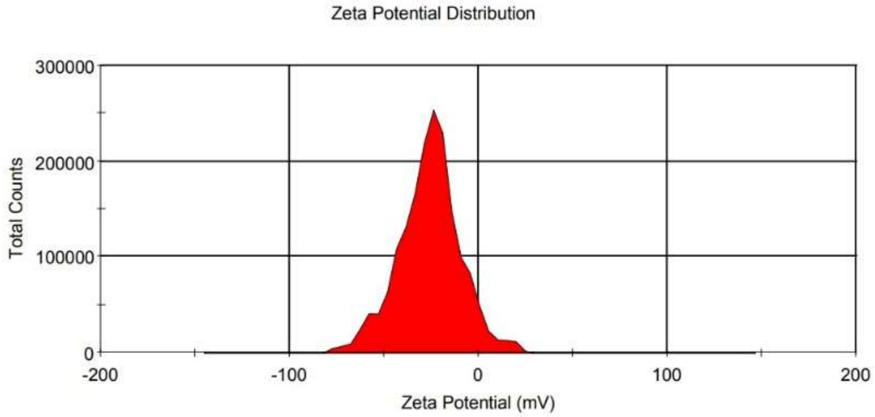

Figure S2. The zeta potential distribution of FOA nanoparticles
